# Inhibition of airway surface fluid absorption by cholinergic stimulation

**DOI:** 10.1038/srep20735

**Published:** 2016-02-05

**Authors:** Nam Soo Joo, Mauri E. Krouse, Jae Young Choi, Hyung-Ju Cho, Jeffrey J. Wine

**Affiliations:** 1 The Cystic Fibrosis Research Laboratory, Stanford University, Stanford, CA 94305-2130, USA; 2 Department of Otorhinolaryngology, Yonsei University, Seoul, Korea

## Abstract

In upper airways airway surface liquid (ASL) depth and clearance rates are both increased by fluid secretion. Secretion is opposed by fluid absorption, mainly via the epithelial sodium channel, ENaC. In static systems, increased fluid depth activates ENaC and decreased depth inhibits it, suggesting that secretion indirectly activates ENaC to reduce ASL depth. We propose an alternate mechanism in which cholinergic input, which causes copious airway gland secretion, also inhibits ENaC-mediated absorption. The conjoint action accelerates clearance, and the increased transport of mucus out of the airways restores ASL depth while cleansing the airways. We were intrigued by early reports of cholinergic inhibition of absorption by airways in some species. To reinvestigate this phenomenon, we studied inward short-circuit currents (Isc) in tracheal mucosa from human, sheep, pig, ferret, and rabbit and in two types of cultured cells. Basal Isc was inhibited 20–70% by the ENaC inhibitor, benzamil. Long-lasting inhibition of ENaC-dependent Isc was also produced by basolateral carbachol in all preparations except rabbit and the H441 cell line. Atropine inhibition produced a slow recovery or prevented inhibition if added before carbachol. The mechanism for inhibition was not determined and is most likely multi-factorial. However, its physiological significance is expected to be increased mucus clearance rates in cholinergically stimulated airways.

In human airways, mucociliary and cough clearance are critical components of mucosal innate defense^1^,^2^. Mucus clearance requires an adequate fluid layer lining the airways, and this is maintained by a balance of electrogenic, anion-mediated fluid secretion and Na^+^-mediated fluid absorption, the latter mediated mainly via the epithelial sodium channel, ENaC^1^,^3^. A fundamental question in airway physiology is how airways orchestrate these opposing forces. This question has mainly been explored in cultured surface epithelia, where it has been shown that cells respond to changes in the fluid volume covering them, resulting in a homeostatically controlled depth^4^,^5^. However, in upper airways, submucosal glands provide an additional and powerful component of secretion to the airways^6^, and in all airways, the rate of mucus transport provides another mechanism for adjusting the depth of airway surface liquid (ASL). In this paper, we consider what occurs when airways are stimulated cholinergically, and reconsider early experiments that showed dual actions of cholinergic stimuli on airway epithelia. We propose that in addition to promoting gland secretion, cholinergic inhibition of surface epithelial sodium absorption promotes increased surface fluid volume from the epithelia themselves. Because increased ASL depth leads to increased mucus transport rates^7^,^8^, these coordinated secretory mechanisms will enhance clearance. Thus, increased mucus clearance rate is an additional homeostatic mechanism to restore ASL depth in addition concomitant with its main function of cleansing the airways.

In Ussing chamber experiments with short-circuited epithelia, the Isc across unstimulated airways of most species is dominated by sodium absorption through ENaC, so that large drops in Isc occur when ENaC is blocked. When studying the effects of agonists such as acetylcholine (ACh), most investigators first block ENaC, allowing them to focus on anion secretion. However, Na^+^ absorption by at least some airway surface epithelia is also affected by ACh. Al-Bazzaz and colleagues^9^,^10^, showed a biphasic response to basolateral carbachol in sheep bronchioles. They observed an initial transient Isc increase followed by a long-lasting decrease due to inhibition of Na^+^ transport. They suggested that ACh-induced inhibition of Na^+^ absorption resulted from increased [Ca^2+^]_i_, based on their prior work measuring Isc and ion fluxes across dog trachea, where it was shown that the Ca^2+^ ionophore, A23187, abolished Na^+^ transport in a Ca^2+^-dependent fashion^11^. In excised human bronchi, 0.1 mM ACh caused an Isc decrease, and net Na^+^ absorption was abolished^12^. ACh also induced an electrically silent flow of Na^+^ and Cl^-^, which they inferred was attributed to submucosal gland secretion. Inhibition of ENaC-mediated Isc by increased [Ca^2+^]_i_ was subsequently shown in cultured mouse endometrial cells, where ATP was used to elevate [Ca^2+^]_i_^13^. Airway ciliated cells in humans^14^ and sheep contain muscarinic receptors^15^ (and references therein), which raise [Ca^2+^]_i_ and increase ciliary beat frequency when stimulated.

Airway ENaC activity is influenced in multiple ways, as expected for the key channel that gates the large electro-chemical driving force for Na^+^ influx into the cell, with resulting profound effects on transmembrane ion and fluid transport. Indeed, the hyperpolarization that occurs when ENaC closes is an efficient way to induce anion secretion, because the recycling of Na^+^ requires 4 times more energy than the recycling of Cl^−^16. Accordingly, factors reported to alter ENaC activity include multiple components of the cytosol^17^,^18^ and of the airway surface liquid (ASL), including proteases and anti-proteases^19^,^20^,^21^. Importantly, many of the regulatory factors in ASL are mainly provided by the glands^22^.

Although inhibition of ENaC is the most obvious way to inhibit absorption, there is evidence for other mechanisms. In sheep trachea, Acevedo^23^ observed biphasic Isc responses to ACh with properties like those seen previously in bronchioles. Surprisingly, however, a biphasic Isc response with corresponding increases and decreases in conductance was still observed after permeabilizing the apical membrane with amphotericin and imposing a K^+^ gradient, suggesting that ACh was transiently activating and then inhibiting basolateral K^+^ channels under these conditions.

With this background, we re-examined cholinergic effects on airway mucosal Isc with a view to understanding its generality and physiological significance.

## Results

### Carbachol inhibited ENaC-dependent Isc stimulation in four species

Isc was measured across excised tracheal epithelia of 5 species: humans, sheep, pigs, ferrets, and rabbits. Isc was also measured across planer sheets of cultured primary human bronchial epithelia (HBE) and H441 human small airway cells. The basic procedure was to measure resting Isc and then the Isc response to carbachol added either before or after benzamil. In all tissues except ferret tracheas the resting Isc was inhibited 50~90% by benzamil; in ferrets benzamil inhibited <20% of the resting Isc.

**Figure 1:**
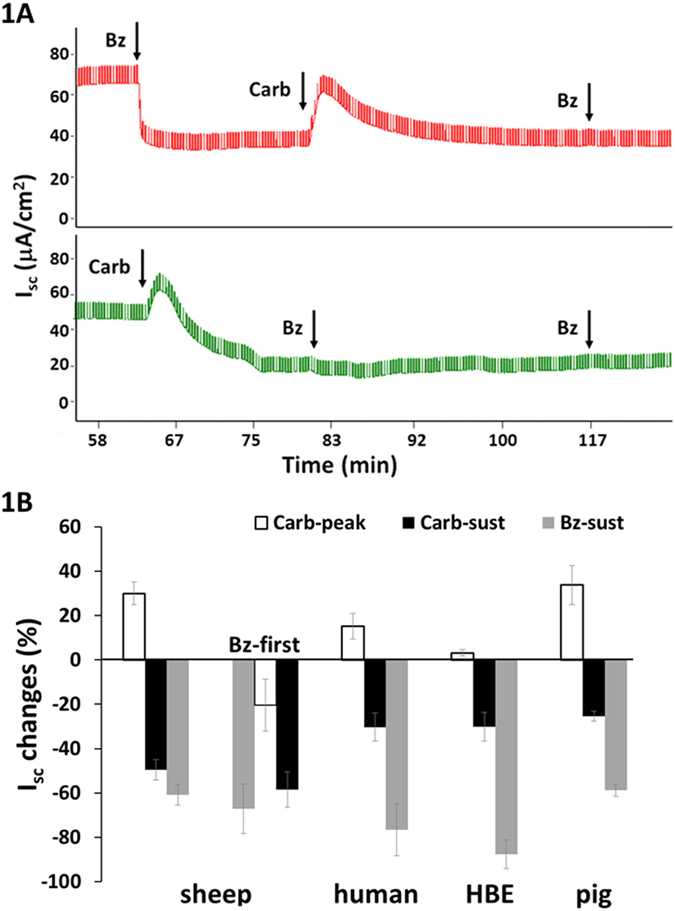
Carbachol inhibits Na^+^ transport across airway surface epithelia. (**A,B**) Ussing chamber Isc responses (1 mV pulse every 20 seconds) from a matched pair of sheep tracheal mucosa to drug additions indicated by arrows. (**A**) Carbachol (100 μM, basolateral, ‘Carb’) followed by 10 μM apical benzamil (Bz) (*top Isc trace*). Drugs given in reverse order: carbachol following benzamil (*bottom Isc trace*). (**B**) Summary of carbachol-induced ENaC inhibition in airways from three different species (n = 4, 13, and 10 human, sheep, and pig, respectively) and primary human bronchial epithelial cells (HBE, n = 9). Carbachol concentration was 1 mM except for sheep (100 μM). Bz-first represents the benzamil treatment preceded carbachol treatment and the bars are showing peak of the transient increase (open bar), sustained (black bar) Isc responses-induced by carbachol, and Isc response-inhibited by benzamil (grey bar).

In sheep, 100 μM basolateral carbachol stimulated a transient increase (t_1/2_ = 88.9 ± 10.6 sec, n = 15) in Isc followed by a sustained decrease (t_1/2_ > 30 min, Fig. 1A, *bottom Isc trace*). When conditions were reversed and benzamil was added first, it produced a sustained decrease in Isc as expected, and addition of carbachol now stimulated only the transient increase in Isc; additional benzamil was without effect (Fig. 1A, *top Isc trace*). Results for sheep are summarized in Fig. 1B and Supplementary Table S.1. Carbachol inhibited the benzamil-sensitive (ENaC-dependent) Isc by 80 ± 3% (n = 15, 13 sheep, P < 0.0001, Fig. 1B), whereas carbachol given after benzamil produced an initial transient increase (167 ± 52%, n = 3 sheep, P = 0.015) followed by a sustained increase in Isc (38 ± 29%, n = 3 sheep, n.s. P = 0.14) (Fig. 1B). Neither of these increases were sufficient to restore the original I_sc_.

Basolateral carbachol also inhibited ENaC-dependent (benzamil-sensitive) Isc in pig, human (Fig. 1B), some ferret tracheas (Supplementary Figure 1C) and in primary HBE cultures (see below). Apical carbachol had no consistent effect in any of these preparations (Supplementary Figure 1B). Carbachol inhibition of sheep and pig airways occurred with concentrations as low as 1 μM, but inhibition at that concentration developed slowly, making it difficult to study. In sheep, 100 μM carbachol rapidly and reliably induced inhibition; in the other species 1 mM carbachol was required to consistently inhibit ENaC-dependent Isc, and in ferrets even that concentration gave inconsistent results. (Note that ferret experiments were done in the presence of cartilage, and ferrets also had the smallest response to benzamil, see below).

In human large airways 76.7 ± 11.7% of the Isc was benzamil-sensitive, and carbachol inhibited 39 ± 3.3% of that Isc (n = 4 human subjects). In pig tracheas 58.8 ± 2.6% of the Isc was ENaC-dependent and carbachol inhibited 44.2 ± 4.7% of that Isc (n = 10 pigs). In ferret tracheas only 17.3 ± 4.2% of Isc, was inhibited by benzamil (n = 32, 16 ferrets). When carbachol was added first, it inhibited the Isc in only 3 of 8 ferrets by 36 ± 20.5% (Supplementary Figure 1C).

### Carbachol inhibited ENaC-dependent Isc in primary cultures of HBE cells

**Figure 2:**
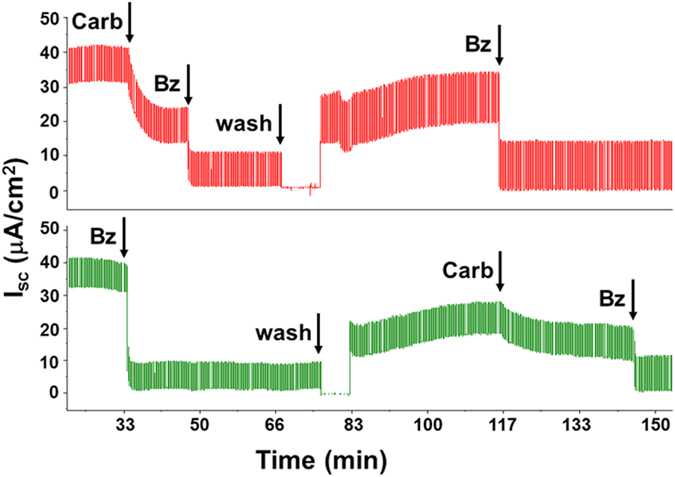
Carbachol inhibition of Isc in primary cultures of human bronchial epithelial cells. Top trace: ENaC-dependent I_sc_ is partially inhibited by 1 mM carbachol and reduced to zero with 10 μM benzamil. Both kinds of inhibition were partially reversed by washing. Bottom trace: all Isc was inhibited by benzamil but not by carbachol. Unlike animal tracheas, an initial transient Isc increase to 1 mM carbachol was either small or absent in HBE cells (n = 9 monolayer inserts).

Stimulation of HBE cells with 1 mM carbachol produced an Isc drop in all monolayer HBE cell inserts (n = 6, Figs 1B and 2 and Supplementary Table S.1). The initial transient increase in Isc was lacking or small. (The transient increase in human airways was also small, see below). In contrast with explanted airways, benzamil subsequently caused a second large drop in Isc (*top Isc trace*) to ~zero Isc, and when benzamil was added first it almost abolished the Isc (87.4 ± 6.9%, n = 3), again in contrast with explanted airway tissues, where ~30% of the original Isc remained after benzamil. Isc recovered slowly after wash out to 60~70% of the Isc which could be inhibited with repeated benzamil (Fig. 2, *top Isc trace*). If benzamil was added first (Fig. 2, *bottom Isc trace*), the subsequent washout increased Isc and part of the recovered Isc was sensitive to 1 mM carbachol. To our knowledge this is the first demonstration of carbachol-induced inhibition of primary HBE cells.

### Inhibition was unchanged after reducing anion transport

**Figure 3:**
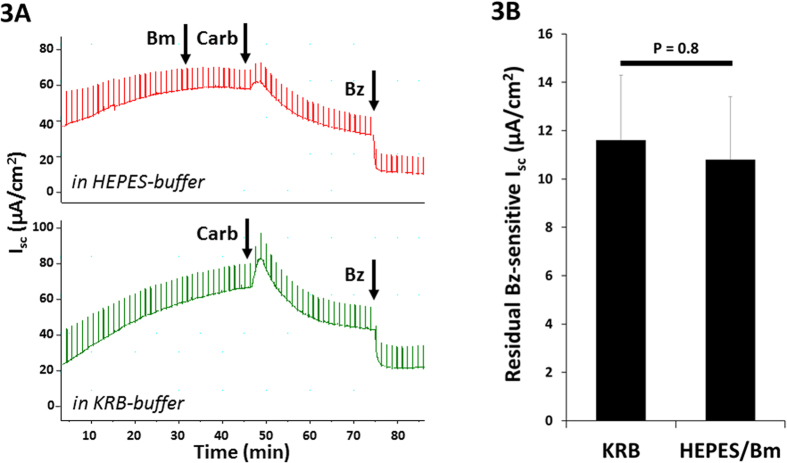
Na^+^ absorption but not anion secretion was inhibited by cholinergic stimulation. **(A)** (*top trace,* anion transport inhibitors present*, bottom trace,* control buffer) In sheep trachea, ENaC-dependent Isc inhibition by carbachol persisted after anion secretion was blocked by 100 μM basolateral bumetanide (Bm) plus HCO_3_^−^ replacement by HEPES with pure O_2_ supply. The transient Isc increase by carbachol was greatly reduced in the bumetanide and HEPES condition. (**B**) Average magnitude of residual benzamil-sensitive Isc in sheep airways after carbachol; control (KRB, Krebs-bicarbonate) vs. HEPES/Bm buffers (n = 6 each, from 4 sheep).

The decline in Isc following carbachol might partially result from inhibition of anion secretion, although that interpretation is not consistent with the small increases in I_sc_ seen when carbachol was added after benzamil. To further explore a role for anion secretion, we stimulated sheep trachea with carbachol in normal Krebs-Ringer bicarbonate (KRB) buffer and in a buffer designed to block anion transport (HEPES for replacing HCO_3_^−^ and 100 μM bumetanide to block, Na^+^/K^+^/2Cl^−^ cotransporter, NKCC). These conditions reduce carbachol-stimulated gland secretion by ~90%^24^. No significant difference in carbachol-induced inhibition of the ENaC-dependent Isc was observed when anion transport was reduced by bumetanide + HEPES (the residual ENaC-dependent ENaC Isc: 11.6 ± 2.7 in KRB and 11.4 ± 2.6 μA/cm^2^, P = 0.8, n = 6 from 4 sheep), while the transient Isc increase was greatly reduced in HEPES + bumetanide buffer (14.7 ± 2.4 in KRB and 7.8 ± 2.8 μA/cm^2^ in HEPES + Bm) (Figure 3).

### Carbachol inhibition of Isc is mediated by muscarinic receptors

**Figure 4:**
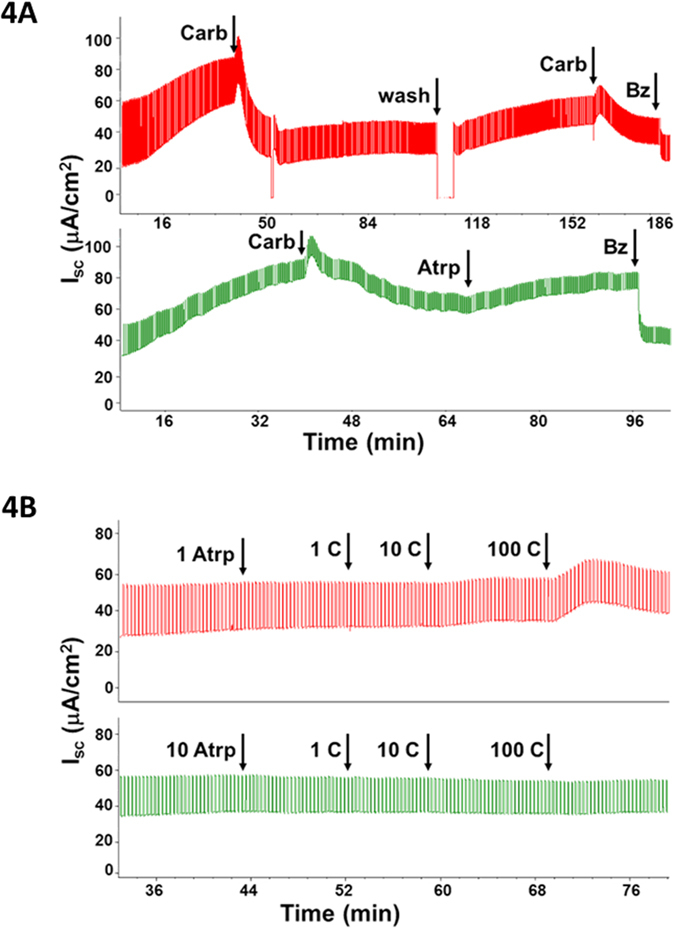
Carbachol inhibition of Isc is mediated by muscarinic receptors. (**A**) Carbachol (100 μM) inhibition of Isc in sheep trachea recovered slowly after washout (*top Isc trace*) and after application of atropine (Atrp, 10 μM, basolateral, *bottom Isc trace*). Inhibition could be repeated (*top Isc trace*). (**B**) Dose-dependent block of cholinergic effects on Isc by atropine. Sheep tracheas treated with 1 or 10 μM atropine prior to 1 to 100 μM carbachol. Both the initial increase and the later decrease in Isc are blocked. Atropine block was observed in 6 experiments with sheep (n = 3) and pigs (n = 2, Supplementary Figure 2A).

Carbachol, like acetylcholine, stimulates both muscarinic and nicotinic receptors. Furthermore, the high concentrations needed to see inhibition of Isc in some preparations raised a concern about off-target effects. To determine if Isc inhibition was mediated by muscarinic receptors, we compared effects of the muscarinic antagonist atropine and the nicotinic antagonist hexamethonium bromide on carbachol inhibition of ENaC–dependent Isc. When atropine was added near the maximum of carbachol-induced inhibition it led to recovery with a time course similar to that seen after wash-out of carbachol (Fig. 4A). When atropine (1 or 10 μM) was added basolaterally before carbachol, it abolished the Isc decreases to carbachol (Fig. 4B) (n = 4 sheep and 2 pigs). In contrast, hexamethonium bromide up to 100 μM had no effect on carbachol-induced inhibition (Supplementary Figure 2A), and nicotine (1 mM, basolateral) failed to alter Isc in pig or sheep tracheal mucosa (3 pigs and 2 sheep, Supplementary Figure 2B).

### Apical ATP inhibited ENaC-dependent Isc, but was not required for carbachol-induced inhibition

**Figure 5:**
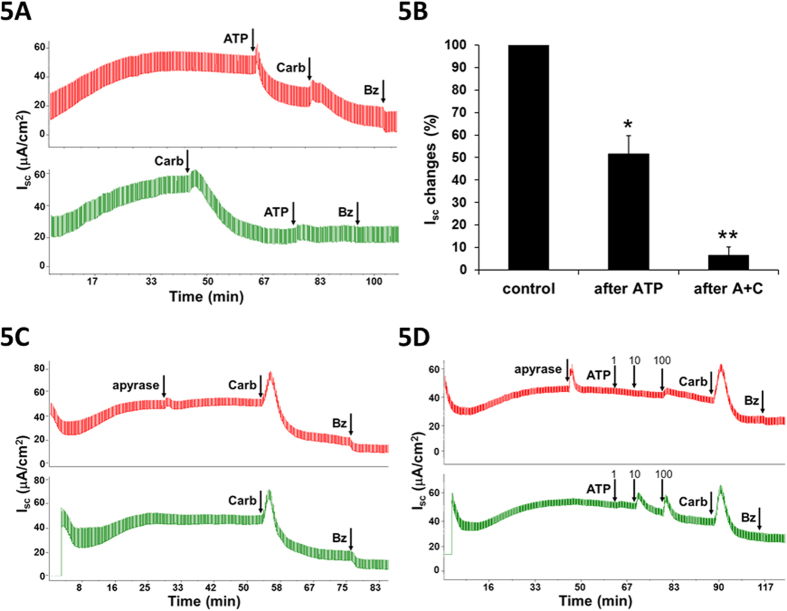
Apical ATP is not required for carbachol-induced inhibition of Isc. (**A**) Inhibition of Isc across sheep trachea by 100 μM apical ATP before (*top trace*) and after (*bottom trace*) 100 μM basolateral carbachol. Inhibition of Isc by ATP is much smaller after carbachol treatment. Benzamil (10 μM) was applied apically. (**B**) Summary of ATP results in sheep airways (n = 3); *,**significantly different from control, “after,” P < 0.05 and P < 0.005, respectively. (**C**) In sheep trachea, apyrase pretreatment (10 U/ml, apical) had no effect on carbachol induced inhibition of ENaC-dependent Isc. (**D**) By contrast, in sheep trachea apyrase (*top trace*) abolished ATP induced inhibition of ENaC-dependent Isc (shown without apyrase in *bottom trace*). Representative traces, n = 6 experiments from 4 sheep.

In several systems^13^,^18^,^25^,^26^, apical ATP inhibits amiloride-sensitive Isc. To investigate the possibility that carbachol-induced inhibition might be mediated by release of ATP, sheep tracheas were pretreated with 100 μM ATP before carbachol addition. As previously described in other systems, ATP decreased the ENaC-dependent Isc. However, subsequent addition of 100 μM carbachol produced an additional decrease in Isc (Fig. 5A, *top Isc trace*). When ATP was added after carbachol-induced inhibition had plateaued, it produced no further inhibition (Fig. 5A, *bottom Isc trace*). Summary data (Fig. 5B) show that ATP alone reduced ENaC-dependent Isc by 48.4 ± 8.1% (n = 3 sheep, P = 0.016) and subsequent carbachol addition almost abolished the remaining ENaC–dependent Isc (P = 0.001). These results show that carbachol inhibits ENaC-dependent Isc more effectively than does ATP, but do not eliminate the possibility that ATP release is a component of carbachol-induced inhibition. To evaluate that possibility we added 10 U/ml apical apyrase (which catalyzes the hydrolysis of ATP), to sheep and pig airway mucosa (n = 7 from 4 sheep and one pig) prior to adding carbachol or ATP. Apyrase pretreatment abolished ATP-induced inhibition but had no effect on the carbachol-induced inhibition (Figure 5C and D). Thus, if ATP is released into the ASL by carbachol its inhibitory effects are occlusive with other inhibitory mechanisms.

### Carbachol failed to inhibit Isc in rabbit trachea and in the human H441 cell line

**Figure 6:**
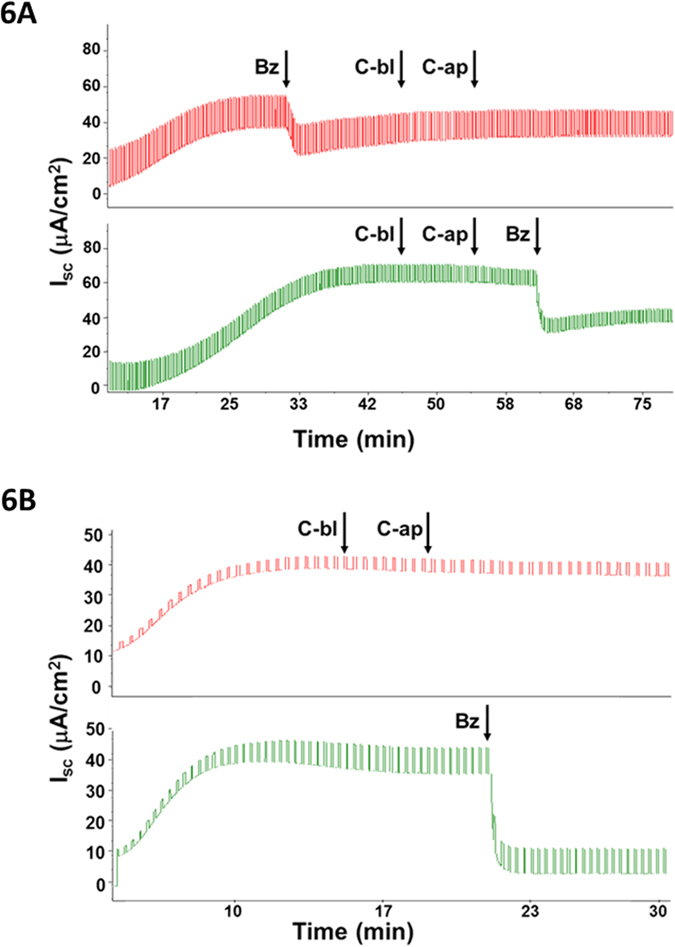
Carbachol did not inhibit ENaC-dependent Isc in rabbit airways or H441 cells. (**A**) Rabbit tracheal preparations were treated with 100 μM carbachol basolaterally (C-bl) and apically (C-ap) after (*top trace*) and before (*bottom trace*) 10 μM apical benzamil (Bz) as indicated by arrows. Representative Ussing Isc records from n = 3 rabbits. (Apical ATP (100 μM) did inhibit ENaC-dependent Isc, see Supplementary Figure 3A). (**B**) H441 human small airway Clara epithelial cells failed to respond to 1 mM carbachol applied to both sides of the filter (*top trace*), in spite of having a large ENaC-dependent Isc (*bottom trace*). Representative traces from 6 H441 cell inserts.

In rabbit tracheas ~50% of total Isc was benzamil-sensitive (ENaC-dependent) prior to stimulation (n = 3 rabbits). When rabbit tracheal mucosa was treated with 100 μM carbachol either to basolateral or apical side before or after benzamil, it produced no change in the Isc (Fig. 6A, n = 4 from 3 rabbits). This lack of responsiveness by rabbit tracheas is consistent with an earlier report that studied excised tracheas^27^. However, *cultured* rabbit tracheal cells responded to carbachol with *increased* I_sc_ and [Ca^2+^]_i_^28^. We confirmed that rabbit tracheal Isc was inhibited by apical ATP (Supplementary Figure 3A), as first shown by Iwase and colleagues^25^.

H441 cells are human small airway cells that express functional ENaC. H441 cells developed robust ENaC-dependent Isc (~93% of their starting Isc: 38.5 ± 4.5 to 2.8±0.8 μA/cm^2^, n = 7 inserts) but they did not respond to basolateral or apical application of 1 mM carbachol (Fig. 6B, n = 6 inserts). Apical ATP produced no inhibition, instead causing a sustained increase in Isc of 6 or 12 μA/cm^2^ (n = 2 inserts, Supplementary Figure 3B, *top Isc trace*). However, ENaC-dependent Isc was inhibited by 4–16 μA/cm^2^ by thapsigargin, which elevates [Ca^2+^]_i_. (Supplementary Figure 3B, *bottom Isc trace*, n = 2 inserts).

These observations indicate that rabbit tracheal and H441 cells also have mechanisms for inhibiting ENaC-dependent Isc, but rabbit tracheal cells either lack muscarinic receptors or fail to couple them to pathways that inhibit the ENaC-dependent Isc, while H441 cells lack mechanisms to couple either muscarinic or purinergic receptors to inhibition of ENaC-dependent Isc.

### Proteases and anti-proteases influenced Isc in H441 cells and airway mucosa

We previously proposed that airway submucosal glands could participate in the regulation of ENaC activity by secreting ENaC regulating factors, such as serine protease inhibitors, onto the airway surface when glands are stimulated^21^,^29^. In our present study we found that the serine protease inhibitor aprotinin significantly reduced ENaC-dependent Isc in H441 cell monolayers when 30 μg/ml was applied apically (control: from 48.8 ± 3.6 to 41.2 ± 2.4 and aprotinin treated: from 47.2 ± 3.8 to 21.2 ± 2.3 μA/cm^2^, P = 0.001, n = 4 inserts). Trypsin partially counteracted carbachol-induced inhibition in pig and sheep airways (Supplementary Figure 3C and D). These results are consistent with our earlier interpretations^21^,^29^ and other reports^19^,^30^.

### Phospholipase-C activation was not required for carbachol-induced inhibition

A phospholipase-C pathway has been implicated in the inhibition of ENaC by flagellin^31^. To determine if cholinergic inhibition of ENaC requires PLC, we tested carbachol-induced inhibition ± the PLC inhibitor, U73122, in sheep and pig tracheas. Apical + basolateral U73122 (10 μM) had no effect on carbachol inhibition of ENaC-dependent Isc (Supplementary Figure 4A).

### CFTR activation with forskolin did not affect carbachol inhibition of ENaC-dependent Isc

CFTR interactions with ENaC have been controversial^3^,^32^. To look for possible interactions, we activated or inhibited CFTR prior to carbachol stimulation and noted effects on ENaC-dependent Isc. The residual ENaC-dependent Isc after carbachol was not significantly different in the presence (2.7 ± 0.6 μA/cm^2^) or absence (2.8 ± 0.5 μA/cm^2^) of 10 μM forskolin pre-treatment (n = 5, 4 sheep, Supplementary Figure 4B).

## Discussion

### Main findings

Cholinergic stimulation causes both gland secretion and increased rates of mucociliary clearance (MCC)^7^,^8^. Because mucus clearance rates increase as the depth of ASL increases^1^,^2^, increased MCC after cholinergic stimulation could result simply from increased ASL depth following gland secretion. This effect will be counterbalanced by absorption, which in cultured epithelial cells increases as fluid depth increases^1^,^2^. This is a means of eventually restoring conditions to the basal state, but during the need for rapid clearance we hypothesized that the effects of glandular secretion on MCC could be amplified by switching the surface epithelia from fluid absorption to fluid secretion—indeed in one system this has been shown directly by inhibiting ENaC with benzamil^7^. How is this normally accomplished *in vivo*? *The main point of this paper is that long lasting inhibition of ENaC-dependent fluid absorption is the most important factor in the switch of surface epithelia from absorption to secretion*.

In the present experiments we explored the generality and physiological significance of cholinergic inhibition of ENaC-dependent Isc by testing the effects of cholinergic stimulation on Isc in seven types of airway preparations that display ENaC-dependent Isc. Of these, five also displayed cholinergic inhibition of ENaC-dependent Isc. The exceptions were rabbit trachea and the H441 human small airway cell line; we suspect but did not establish that these cells lack muscarinic receptors.

### Comparison with apparently contradictory results

Some prior studies do not report cholinergic inhibition of ENaC-dependent Isc. A previous study of dog tracheas found no effect of acetylcholine on inhibition of inward Na^+^ current, and only a slight inhibition of Na^+^ flux^33^. Dogs, like rabbits, may lack this response, but the concentration of ACh was less and the measurement duration shorter than we used, so dog tracheas will need to be re-examined using comparable conditions to establish this point.

In an elegant study, results opposite to those reported here were found in cultured rat alveolar type II cells, where carbachol *activated* ENaC activity^34^. Different actions of muscarinic agents in different cell types are not contradictory. Indeed, if viewed functionally, they are complementary actions to remove fluid from the airways. Fluid removal from the lung alveoli by A2 cells occurs by direct fluid absorption, which was increased by systemic carbachol^34^. However, for the airways, with their vastly reduced surface area compared to alveoli, the mechanism for fluid removal is primarily via mucociliary clearance, which is accelerated when ENaC-dependent absorption is inhibited^7^ (Joo, unpublished observation with *ex vivo* ferret tracheas). Thus, paradoxically, inhibition of airway epithelial absorption will increase the rate of fluid (mucus) clearance from the airways to restore ASL depth. As mentioned above, this mechanism can’t operate in closed, cell culture systems, where fluid absorption is the only means for decreasing depth.

### Mechanisms of carbachol inhibition of ENaC-dependent Isc: apical mediators

An efficient way to couple gland secretion to inhibition of epithelial surface absorption would be to have the glands themselves secrete factors to inhibit ENaC^22^ (and references therein). This hypothesis is supported by evidence that ENaC is inhibited by multiple factors in the ASL^17^,^18^,^19^,^20^, many of which are abundant in glands^6^,^21^,^22^,^29^, For example, the BPI fold protein SPLUNC1 inhibits ENaC activity and fluid absorption of airway surface epithelia and has been proposed as the major endogenous ENaC regulator of upper airways surface epithelia^20^. BPI fold proteins are most highly expressed in submucosal glands in the upper airways^35^,^36^ and are the most abundantly detected innate defense proteins in pure mucus secreted from human airway submucosal glands^22^. In Ussing chambers the ASL is diluted extensively so that fluctuations in ASL soluble factors are unlikely to play a significant role in the responses we observed. However, to show that such factors can in principle participate in the cholinergic response we showed that the antiproteases, aprotinin decreased Isc in H441 cells and the protease trypsin increased it in sheep and pig airway. It remains necessary to demonstrate directly that stimulating glands inhibits benzamil-sensitive Isc and if so, to assess the importance of their role.

The logic is compelling that glandular secretions increase mucus clearance rates both by direct secretion of mucus and by switching the surface epithelia to a secretory mode by inhibiting ENaC-dependent fluid absorption.

### Mechanisms of carbachol inhibition of ENaC-dependent Isc: basolateral mediators

In spite of controversy about whether epithelia are innervated^37^,^38^,^39^, human^14^ and sheep airway ciliated cells^15^ express muscarinic receptors, and 4 of the 5 species tested plus cultured HBE cells responded to the muscarinic agonist carbachol. We do not know the cellular mechanisms that intervene between activation of muscarinic receptors and inhibition of ENaC-dependent Isc, but the combined results of many investigators added to our own make it clear that multiple mechanisms must be involved. It needs to be emphasized that there is no direct evidence from any study that ENaC activity of tracheal epithelial cells is decreased by cholinergic stimulation, although that is the assumption. ENaC *P*_*O*_ can be decreased in tracheal cells by ATP^17^ and increased in cultured A2 cells by muscarinic stimulation^34^. But even in the careful analysis of A2 cells only the early links in the pathway from muscarinic receptors were described^34^.

An alternative or additional mechanism is the inhibition of basolateral K^+^ channels^23^. Because the anion secretion that continues in the presence of carbachol (Fig. 1, Supplementary Figures 5A and B) is sensitive to the anion channel inhibitor, niflumic acid, this inhibition can only be partial, since some residual K^+^ conductances are necessary to sustain the anion secretion. In addition to these potential mechanisms, ENaC-dependent Isc is modulated by the membrane potential, so that cholinergic activation of anion conductances will reduce the driving force for Na^+^ entry independent of any effect on ENaC number or *P*_*O*_. In sum, detailed patch clamp and cell biological analyses of carbachol-induced inhibition of ENaC-dependent Isc are warranted and feasible now that we have demonstrated the phenomenon in cultured cells HBE cells, but our focus has been on the physiological relevance, not the mechanism.

### Limitations of this study

In addition to the limitations discussed above, methods beyond I_sc_ studies will be required to establish mechanisms. The high concentrations of carbachol that were required to see ENaC-dependent Isc are puzzling, because it is unlikely that these occur physiologically, even as part of strong parasympathetic activation during the lung defense reflex^40^. This is especially so since direct cholinergic synapses on airway epithelia have not been described and the source of ACh is likely to be more diffuse. We are confident that these are not off-target effects, because atropine eliminated the high dose carbachol effects (Supplementary Figure 2A-*bottom Isc trace*). For cholinergic mediation of gland secretion, co-transmitters such as VIP produce markedly synergistic effects^6^, so it is possible that our experiments lack co-factors needed to amplify inhibition by carbachol. Also, at concentrations of carbachol below threshold for most preparations, those that responded showed a very slowly developing inhibition that eventually reached a level similar to that produced rapidly by much higher concentrations (Supplementary Figure 6). The variability and very slow development of responses to lower concentrations precluded its further study, but it remains possible that intact airways might exert tonic control of fluid absorption with low levels of acetylcholine (and perhaps with other co-transmitters).

### Physiological relevance: parasympathetic coordination of optimal mucus clearance

This work was influenced by earlier work showing that ACh increased ciliary beat frequency via an M3 receptor present in airway epithelial cells^15^, by evidence that carbachol stimulated gland secretion and mucociliary clearance rates in pig^7^ and ferret^8^ tracheas, and that benzamil speeded MCC rates after anion transport had been diminished^7^. We hypothesize that parasympathetic stimulation *in vivo* will also inhibit ENaC-dependent, Na^+^-mediated fluid absorption, and help switch the balance of epithelia transport to net secretion, thus augmenting gland secretion and increased ciliary beat frequency to speed mucus clearance. If this interpretation is correct, it suggests caution in the use of inhaled anti-cholinergic agents to treat patients with obstructive pulmonary diseases, since these might actually slow MCC^41^.

## Materials & Methods

### Airway tissue procurement

Human tracheal scraps from donors were obtained from lung transplants harvested less than 2 h post-mortem at Stanford Hospital. The protocol for handling human airway tissues was approved and informed consent was obtained from all participants prior to the study (Stanford University-IRB protocol#: 11638). Postmortem (<1 hr) tracheas from young male and female *Yorkshire* pigs (30~50 kg), young male *Suffolk-Rambouillet* sheep (~70 kg), *New Zealand White* rabbits at the age of 5~6 months, and 3~10 months old *M. Putorius* ferrets were obtained from the animal facility at Stanford University after acute experiments unrelated to our studies. All protocols for handling animal tissues at Stanford were approved by Administrative Panel on Laboratory Animal Care (Stanford’s Institutional Animal Care and Use Committee: IACUC protocol#: 10048). Methods for both human and animal tissues were carried out in accordance with approved guidelines. Harvested tissues were placed in cold PhysioSolTM (Hospira, IL) after removal for transport to the laboratory and then transferred to ice-cold Krebs Ringer bicarbonate (KRB) buffer gassed with 95% O_2_ and 5% CO_2_ and kept until use. The KRB buffer composition was 115 mM NaCl, 2.4 mM K_2_HPO_4_, 0.4 mM KH_2_PO_4_, 25 mM NaHCO_3_, 1.2 mM MgCl_2_, 1.2 mM CaCl_2_, and 10 mM Glucose (pH 7.2 at room temperature) adjusted to ~290 mosM with a Wescor vapor pressure osmometer. The Krebs buffer contained 1.0 μM indomethacin to minimize endogenously generated prostaglandins during tissue preparation. Where HCO_3_^−^-free bathing buffer solution was used, HCO_3_^−^ was replaced by HEPES, 4-(2-hydroxyethyl)-1-piperazineethanesulfonic acid, and gassed with 100% O_2_. Tracheal mucosa for Ussing chamber experiments were carefully dissected from underlying cartilage while those from rabbits and ferrets were used intact without dissecting cartilage and connective tissues and only cartilaginous portions of trachea were used.

### Cell culture

The human airway surface epithelial cell line^21^, H441, was maintained in a T_25_ tissue culture flask containing a 1:1 mixture of Dulbecco’s modified Eagle’s medium and Ham’s F-12 nutrient mixture supplemented with 10% fetal bovine serum (Sigma, MO), 100 μg/ml streptomycin, 100 units/ml penicillin, and 2 mM glutamine at 37° C in a humidified atmosphere containing 5% CO_2_. Cells were passaged once at 7~10 days and seeded on HPC (human placental collagen, Sigma)-coated filters (Snapwells, Corning-Costar). To provide an optimal air-interface cultures, the serum medium was switched with 2% Ultroser-G (BioSepra, Cergy, France), a serum substitute, containing medium in cells grown on filters 3~4 days after seeding. Human airway primary cell cultures used in this study were generously provided by Philip Karp, Jonathan Widdicombe and Walter Finkbeiner.

### Electrophysiology

Human and animal airway tissue preparations and cultured cells on filters were mounted in EasyMount Ussing chambers (Physiologic Instruments, CA) with exposed surface areas of 0.07~1.13 cm^2^ depending on the preparation, and were bathed in KRB buffer at 37 °C with a continuous 95% O_2_ and 5% CO_2_ gas supply, except for HCO_3_^−^-free buffer solutions which were gassed with pure O_2_. Transepithelial short-circuit current (Isc) was measured using a VCC-600 voltage clamp (Physiologic Instruments, CA) and Isc data were obtained and displayed with PowerLab Chart4 software (ADInstruments, CA). Total tissue conductance was calculated by applying Ohm’s law to the Isc deflection resulting from 1 mV pulse across the tissues/cell cultures every 20 sec during the experiment (Supplementary Table S.1).

### Reagents

Chemicals were obtained from Sigma/Aldrich (St. Louise, MO) and were made fresh or maintained at −20 °C as aliquots of stock concentrations. Stock solutions of carbachol, ATP, apyrase (ATP diphosphohydrolase), aprotinin, trypsin, and hexamethonium bromide were made in sterilized deionized water and bumetanide, an inhibitor of Na^+^/K^+^/2Cl^−^ transporter in alkaline solution while benzamil, CFTRinh172, GlyH101, thapsigargin, U73122 (a phospholipase C, PLC, inhibitor), and atropine were dissolved in dimethyl sulfoxide (DMSO, Me_2_SO). Carbachol-induced Isc responses were indistinguishable from those produced by acetylcholine (Supplementary Figure 1A). Therefore, we used carbachol (a metabolically non-hydrolyzable analog) instead of acetylcholine to avoid potential variability that could be caused by differing levels of acetylcholinesterase across species. Benzamil was chosen because it is a more potent inhibitor of ENaC than amiloride, giving maximal inhibition at 10 μM^42^.

### Statistics

Data are presented as mean ± S.E.M. unless indicated otherwise. Student’s *t*-test was used to compare means of different treatment groups. ENaC-dependent Isc was calculated either by subtracting the starting Isc from the Isc after benzamil (benzamil-first, no carbachol pretreatment) or by subtracting starting Isc from Isc after benzamil (but carbachol treatment- preceded) (Fig. 1B and Supplementary Table 1). Benzamil-sensitive I_sc_ is alternately referred to as ENaC-dependent I_sc_.

## Additional Information

**How to cite this article**: Joo, N. S. *et al.* Inhibition of Airway Surface Fluid Absorption by Cholinergic Stimulation. *Sci. Rep.* **6**, 20735; doi: 10.1038/srep20735 (2016).

## Supplementary Material

Supplementary Information

Supplementary Information
