# Supplementary material for: Inhibition of airway surface fluid absorption by cholinergic stimulation
Source: Sci Rep. 2016 Feb 5;6:20735. doi: 10.1038/srep20735 (PMC4742893; doi:10.1038/srep20735)
Supplement: Supplementary Information [file srep20735-s2.pdf]

Inhibition of Airway Surface Fluid Absorption by Cholinergic Stimulation

Nam Soo Joo, Mauri E. Krouse, Jae Young Choi, Hyung-Ju Cho, and Jeffrey J. Wine

Supplementary Figure S.1

Supplementary Figure S.2

Supplementary Figure S.3

Supplementary Figure S.4

Supplementary Figure S.5

Supplementary Figure S.6

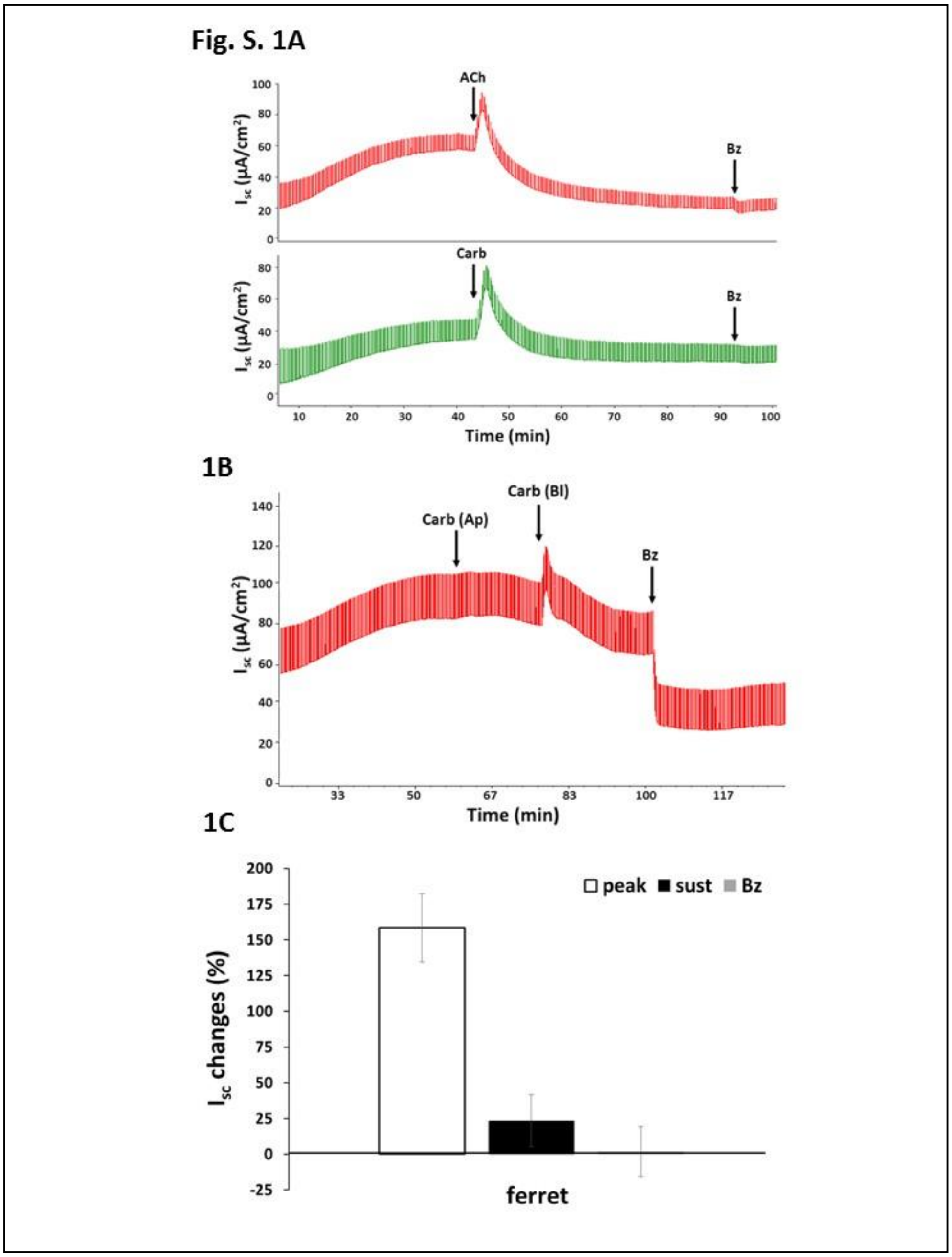

Supplementary Figure S. 1. ENaC Isc inhibition by cholinergic stimulation. (**A**) Identical ENaC inhibition by acetylcholine (ACh) and carbachol (Carb). Acetylcholine produces comparable ENaC inhibition when compared by its non-hydrolyzable analog, carbachol. 100 µM ACh or Carb was added basolaterally in sheep trachea. (**B**) ENaC Isc is inhibited by basolateral (Bl), but not apical (Ap), carbachol treatment. 10 µM carbachol is added apically or basolaterally in pig airways. Representative Isc trace from 3 pig tracheas. (**C**) Summary of carbachol effect on benzamil-sensitive ENaC Isc in ferret airways (n = 8). Unlike sheep, human, and pig airways, carbachol inhibits the Isc in only 3 of the 8 ferrets. Note that 1 mM carbachol induces a larger peak Isc response in ferret airways, when compared by others (**Fig. 1B**).

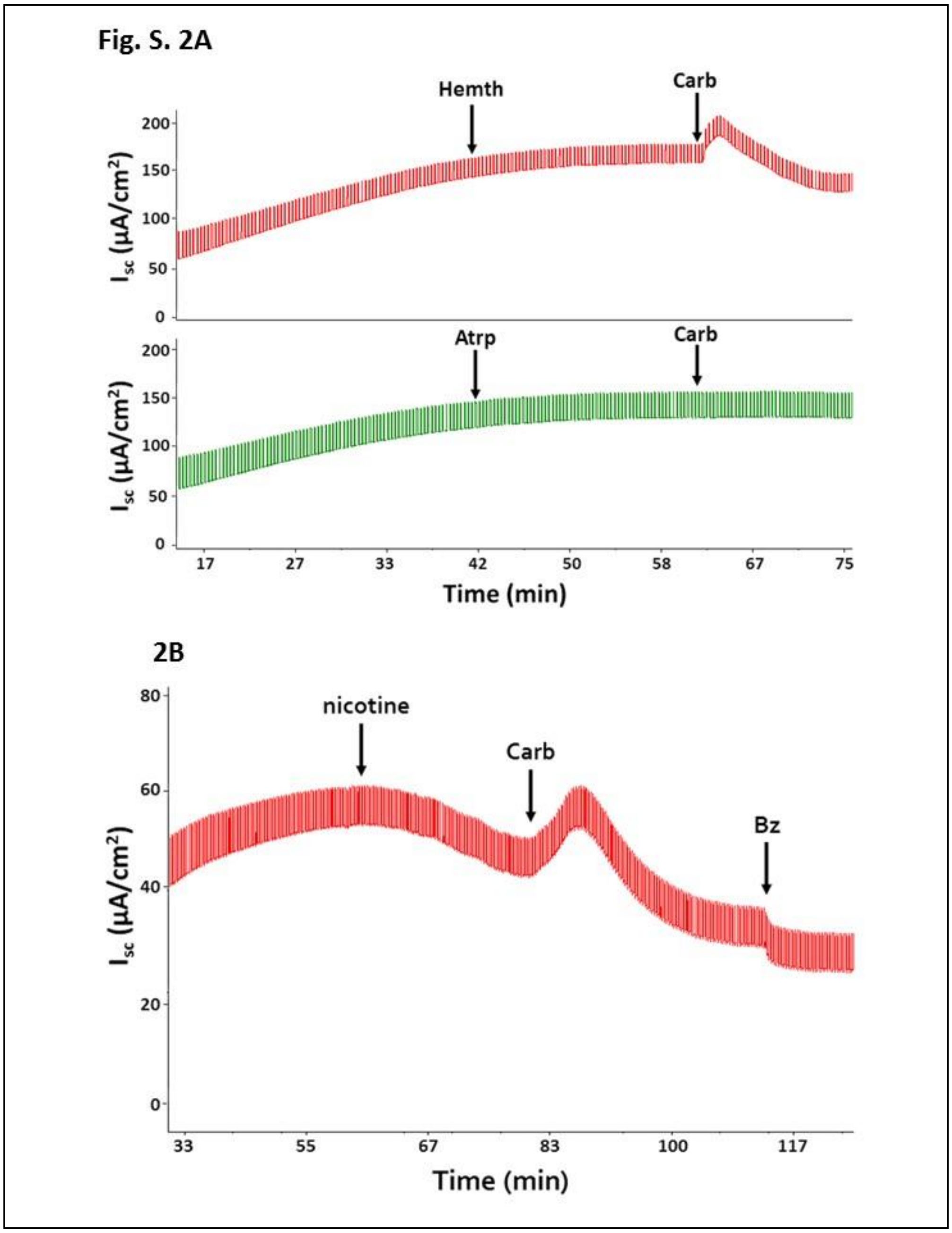

Supplementary Figure S. 2. Lack of hexamethonium bromide effect on ENaC Isc inhibition by cholinergic stimulation. (**A**) Pig tracheas are treated with 100 μM hexamethonium bromide (Hemth, *top Isc trace*) or 100 μM atropine (Atrp, *bottom Isc trace*) basolaterally, prior to 1 mM carbachol (n = 3 pig tracheas). (**B**) Lack of nicotine-induced Isc response. Sheep tracheas are treated 1 mM nicotine basolaterally and followed by 1 mM carbachol and 10 μM benzamil (n = 2 sheep tracheas).

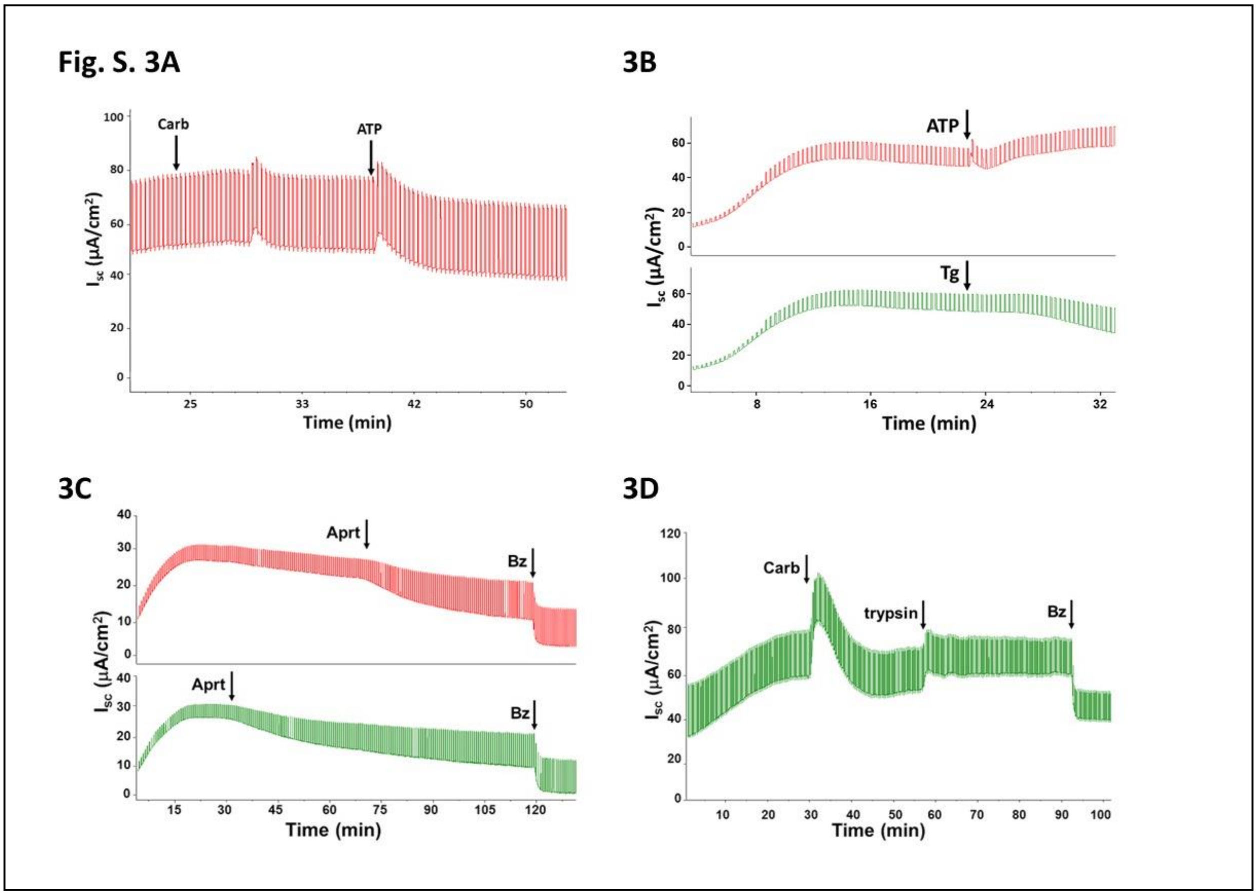

Supplementary Figure S. 3. Effects of ATP, thapsigargin, trypsin, and aprotinin on airway epithelia. (**A**) Presence of benzamil-sensitive ENaC Isc inhibition by ATP in rabbit tracheas. 100 µM basolateral carbachol has no effect but 100 µM apical ATP induces small Isc inhibition in a rabbit trachea. The initial peak response is produced by 1 µM apical and basolateral substance P. (**B**) Apical 100 µM ATP induces a sustained Isc increase (*top Isc trace*, n = 2 H441 inserts), while 1 µM apical and basolateral thapsigargin (Tg) induces ENaC Isc inhibition (*bottom Isc trace*, n = 2 H441 inserts). (**C**) A serine protease inhibitor, 10 µg/ml apical aprotinin, significantly inhibits benzamil-sensitive ENaC Isc (P = 0.001, n = 4 H441 inserts). (**D**) Apical trypsin, a serine protease, counteracts, in part, the ENaC inhibition induced by cholinergic stimulation. Pig tracheal mucosa was treated 1 µg/ml trypsin apically post the basolateral 100 µM Carb. Note ENaC Isc inhibited by carbachol is partially restored by apical trypsin treatment (a representative trace from 3 sheep and two pig tracheas).

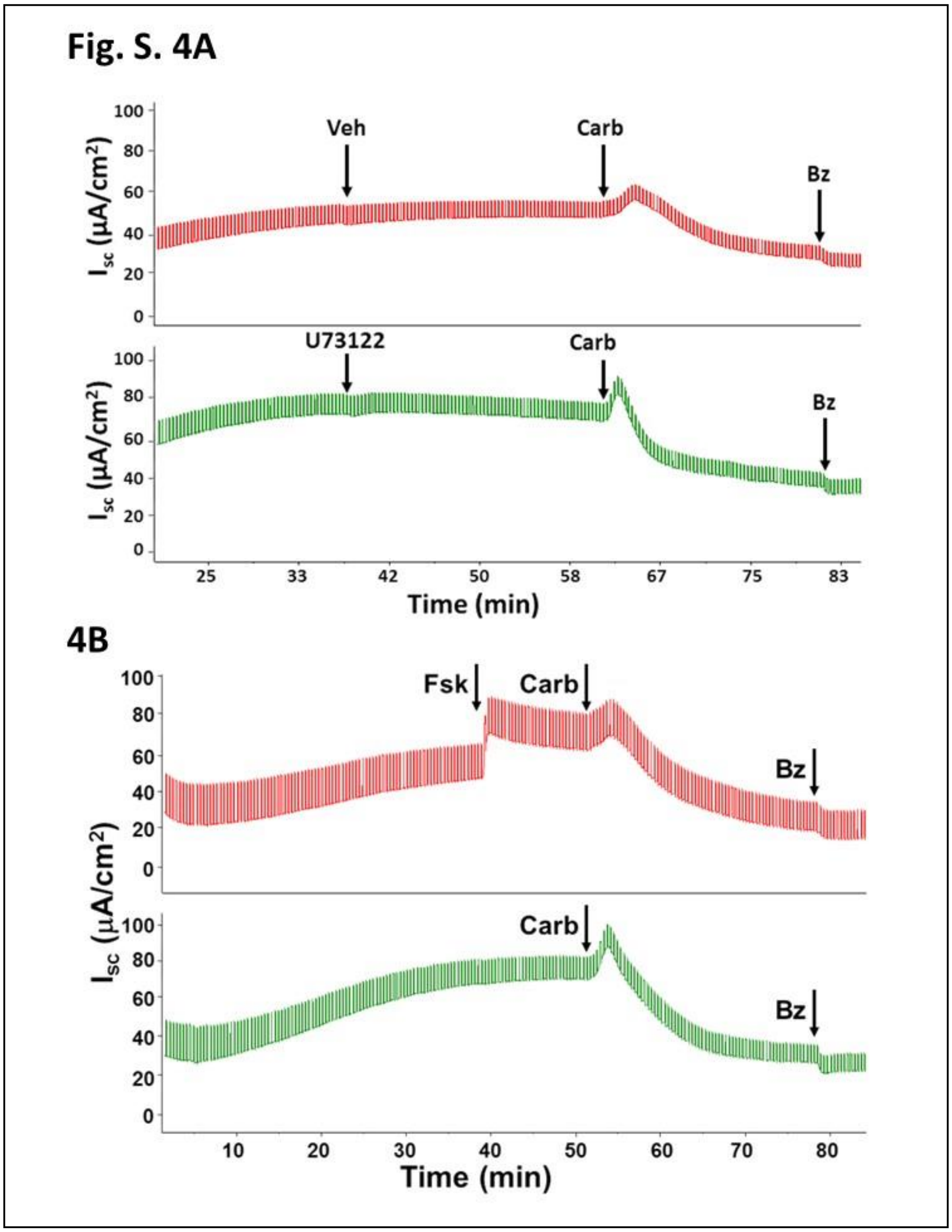

Supplementary Figure S. 4. Effects of U73122 and forskolin on ENaC Isc inhibition by cholinergic stimulation. (**A**) Lack of U73122, a phospholipase-C inhibitor, effect on ENaC Isc inhibition by cholinergic stimulation. Either U73122, 10 µM, or vehicle (Veh, DMSO) was added to both apical and basolateral baths, prior to basolateral 100 µM carbachol treatment, followed by apical benzamil (Bz, 10 µM) addition in sheep tracheal preparations. Representative Isc traces from 2 pigs and one sheep. (**B**) Involvement of CFTR in the ENaC inhibition by cholinergic stimulation. Sheep tracheal mucosa were treated with both apical and basolateral 10 µM forskolin (Fsk) to activate CFTR. Note that the residual benzamil-sensitive ENaC Isc is not

significantly affected by forskolin stimulation prior to basolateral 100 µM carbachol (P = 0.9, n = 4 sheep tracheas).

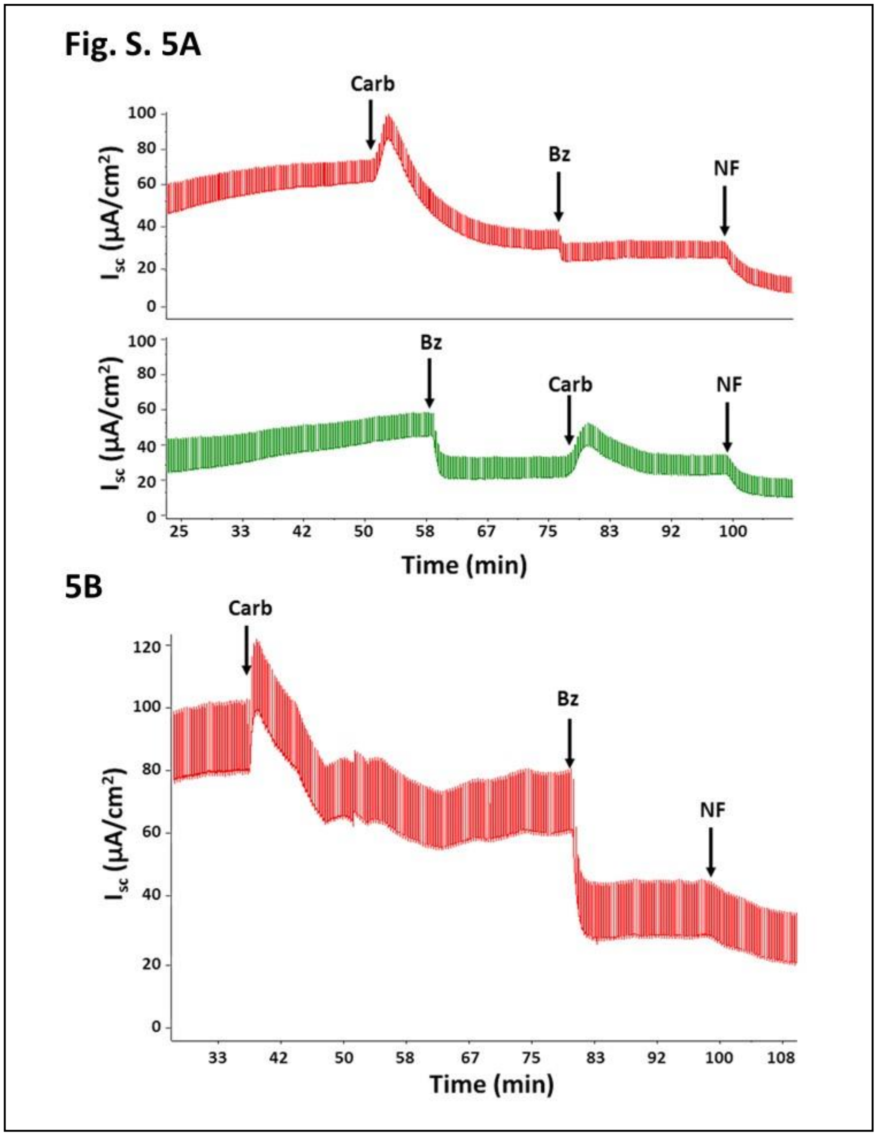

Supplementary Figure S. 5. Carbachol-induced Isc was partially inhibited by niflumic acid. (**A**) Sheep tracheal preparations are treated either with 100 µM basolateral carbachol (Carb) first (*top Isc trace*) or 10 µM apical benzamil (Bz) first (*bottom Isc trace*), prior to 200 µM niflumic acid (NF, an anion channel inhibitor) to the apical and basolateral baths. (**B**) A pig tracheal preparation is treated 100 µM basolateral carbachol, followed by 10 µM apical benzamil, prior to 200 µM niflumic acid to the apical and basolateral baths.

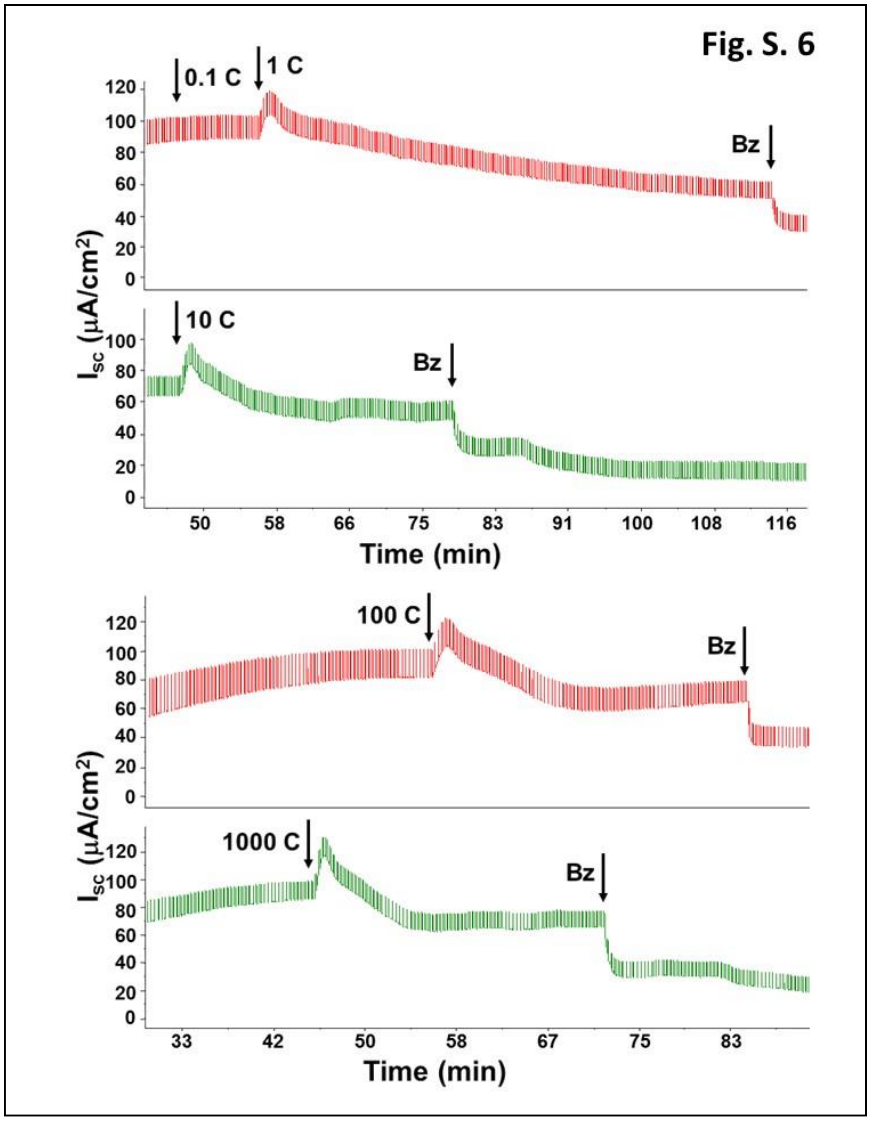

Supplementary Figure S. 6. Time-dependent ENaC inhibition by cholinergic stimulation. ENaC inhibition by cholinergic stimulation appears to be time-dependent rather than dose-dependent when airway tissue preparations are responsive to lower doses of carbachol as shown in pig tracheal mucosa. The carbachol concentrations were 0.1~1000 μM, represented by 0.1 C to 1000 C. Representative Ussing Isc recordings are shown from similar experiments of n = 3 pigs. Note that all workable concentrations of carbachol produce a quite comparable magnitude of peak and sustained Isc responses. The times taken to a point of maximal Isc decrease, however, are shorter as carbachol concentrations increase.
